# Effects of N Fertilizer Sources and Tillage Practices on NH_3_ Volatilization, Grain Yield, and N Use Efficiency of Rice Fields in Central China

**DOI:** 10.3389/fpls.2018.00385

**Published:** 2018-03-22

**Authors:** Tianqi Liu, Jinfeng Huang, Kaibin Chai, Cougui Cao, Chengfang Li

**Affiliations:** ^1^MOA Key Laboratory of Crop Ecophysiology and Farming System in the Middle Reaches of the Yangtze River, College of Plant Science and Technology, Huazhong Agricultural University, Wuhan, China; ^2^Hubei Collaborative Innovation Center for Grain Industry, Yangtze University, Jingzhou, China

**Keywords:** N recovery efficiency, NH_3_ flux, no-tillage, organic N fertilizer, slow-release N fertilizer

## Abstract

Tillage practices and nitrogen (N) sources are important factors affecting rice production. Few studies, however, have examined the interactions between tillage practices and N fertilizer sources on NH_3_ volatilization, nitrogen use efficiency (NUE), and rice grain yield. This study aimed to investigate the effects of N fertilizer sources (no N fertilizer, inorganic N fertilizer, organic N fertilizer alone, organic N fertilizer plus inorganic N fertilizer, and slow-release N fertilizer plus inorganic N fertilizer) and tillage practices (no-tillage [NT] and conventional intensive tillage [CT]) on NH_3_ flux, grain yield, and NUE in the rice field of central China. N sources significantly affected NH_3_ volatilization, as the cumulative volatilization from the treatments of inorganic N fertilizer, organic N fertilizer, organic N fertilizer plus inorganic N fertilizer, slow-release N fertilizer plus inorganic N fertilizer was 4.19, 2.13, 3.42, and 2.23 folds in 2013, and 2.49, 1.68, 2.08, and 1.85 folds in 2014 compared with that under no N fertilizer treatment, respectively. The organic N fertilizer treatment had the lowest grain yield and NUE among all N fertilizer treatments, while slow-release N fertilizer plus inorganic N fertilizer treatment led to relatively higher grain yield and the greatest N use efficiency. Moreover, NT only markedly increased NH_3_ volatilization from basal fertilizer by 10–14% in average compared with CT, but had no obvious effects on total volatilization during the whole seasons. Tillage practices had no significant effects on grain yield and NUE. Our study suggested that the combination of slow-release N fertilizer plus inorganic N fertilizer and NT might be a sustainable method for mitigating greenhouse gas and NH_3_ emissions and improving grain yield and NUE in paddy fields of central China.

## Introduction

Nitrogen (N) is one of the most important nutrients for agricultural systems, and thus N fertilizers are frequently used with the aim to achieve high yields of crop. As the most important cereal crop in China, rice accounts for 18.2% of total cultivated land area in China, and inorganic N fertilizers account for 36.2% of the total chemical N fertilizers used in rice production in the world (Department of Rural Social and Economic Investigation of the National Bureau of Statistics, 2017). However, nitrogen use efficiency (NUE) of the N fertilizers applied in rice production usually falls within the range of 20–40% in China (Xu et al., 2013; Zhang and Zhang, 2013), which not only leads to low rice yields but also causes threat to the environment and human health. The NUE may be ascribed to nitrification, denitrification, NH_3_ volatilization, runoff, and leaching in rice fields (Peng et al., 2006). Therefore, it is highly necessary to optimize the use of N fertilizers to reduce N losses and increase NUE in rice fields in China.

NH_3_ volatilization is an important pathway of N fertilizer loss in paddy fields in China (Xu et al., 2013), which can usually account for 9–40% of the used N fertilizers (Fan et al., 2006). N management involves using an application source, rate, placement and timing that affect NH_3_ emissions (Huang et al., 2016; Zheng et al., 2016). Effective N management, such as using controlled-release N fertilizer and mixture of organic and inorganic N fertilizers or deep N placement (Qi et al., 2012; Chen et al., 2015; Geng et al., 2015; Liu et al., 2015), can more closely match crop N uptake and lower NH_3_ volatilization (Huang et al., 2016), which ensures an adequate amount of N required by the crop to maximize crop yields and NUE. Therefore, great efforts have been made to reduce NH_3_ volatilization and increase NUE through using slow-release N or organic N fertilizers to partly or totally substitute inorganic N fertilizers in paddy fields (Singh et al., 2009; Xu et al., 2013; Huang et al., 2016; Ke et al., 2017; Li et al., 2017). There is growing evidence showing that full or partial substitution of inorganic N fertilizers with slow-release of organic N fertilizers could mitigate NH_3_ emissions and thus increase NUE and rice yields (Chen et al., 2010; Huang et al., 2016; Li et al., 2017). However, other researchers found that crop yields and NUE could be significantly decreased when more inorganic N fertilizers was replaced by slow-release N fertilizers or organic N fertilizers (Bayu et al., 2006; Golden et al., 2009; Yang et al., 2015). Therefore, it is highly necessary to investigate effects of different N fertilizers on NH_3_ emission, NUE, and yields in paddy fields.

As one of conservation tillage practices, no-tillage (NT) has been adopted worldwide due to its advantages in conserving water and soil, reducing input costs, increasing soil organic carbon, and improving crop productivity (Zhang et al., 2014; Pittelkow et al., 2015a,b). In recent years, the NT has been widely implemented in paddy fields in China (Derpsch et al., 2010; Huang et al., 2011; Liang et al., 2016). There is consensus on the effects of NT on NH_3_ volatilization compared with conventional intensive tillage (CT) (Rochette et al., 2009; Zhang et al., 2011; Afshar et al., 2018). It has been well demonstrated that NT could promote NH_3_ volatilization compared with CT due to the improvement of soil urease activity and the presence of crop residues under NT as well as the penetration of a fraction of the fertilizer N into soil shallow cracks under CT (Mkhabela et al., 2008; Rochette et al., 2009). However, the effect of NT on rice grains yields varies considerably (Pittelkow et al., 2015a,b). For example, Gao et al. (2004) reported that the rice grain yield under NT was higher than that under CT in eastern China, possibly due to the improvement of soil physical and chemical properties. Mishra and Singh (2012) also reported similar results, and found that NT resulted in significantly higher yields of rice, wheat and rice–wheat system relative to CT of a dry seeded rice–wheat system on a Vertisol in central India. Panday et al. (2008) observed similar rice grain yields between NT and CT in the northwestern Himalayan region and Zhang et al. (2015) reported that soil tillage did not affect both rice and wheat grain yields on a rice-wheat cropping system of Taihu region in China. Some researchers reported lower rice grain yields under NT relative to under CT (Gathala et al., 2011; Liang et al., 2016). The variations regarding the effects of NT on rice yield may be attributed to the differences in soil properties and field management practices (Gathala et al., 2011; Huang M. et al., 2012; Zhang et al., 2015). Hence, more research is needed to determine the influence of tillage practices on rice grain yields. The interaction between tillage practices and N fertilizer sources is important from the perspective of crop production (Balkcom and Burmester, 2015). However, little is known about the interactions between tillage practices and N fertilizer sources on NH_3_ volatilization, NUE, and rice grain yield. Hence, this study was aimed to investigate the effects of N sources and tillage practices on the above-mentioned parameters in the paddy fields of central China. We hypothesized that N sources significantly affected NH_3_ volatilization, grain yields and NUE, in which inorganic N fertilizers replaced by slow-release N fertilizers or organic N fertilizers could mitigate NH_3_ volatilization and increase grain yields and NUE. We also hypothesized that NT could improve NH_3_ volatilization, and increase grain yields and NUE compared with CT.

## Materials and Methods

### Site Description

The experimental field (29°510′N, 115°330′E) is situated in Wuxue City, Hubei Province, China. The climate of this region is a humid mid-subtropical monsoon climate as described in detail by Zhang et al. (2016). The paddy soil, a type of sandy loam soil, is classified as Gleysol (FAO classification). The rice (*Oryza sativa*, LYP9) and oilseed rape (*Brassica napus*, HS3) varieties were planted. The mean monthly air temperature and rainfall of the experimental site are shown in **Table 1**. The soil properties were described in our previous study (Zhang et al., 2016).

**Table 1:** Mean monthly air temperature (°C) and rainfall (mm) of the experimental site.

Month	2013		2014	
	Mean air temperature	Rainfall	Mean air temperature	Rainfall
June	25.80	226.80	25.72	124.70
July	29.85	30.10	27.46	218.90
August	29.76	65.70	26.20	51.70
September	23.50	64.40	24.34	22.30

### Experimental Design

The field study was conducted from 2013 to 2014. The study included five N fertilizer treatments [no N fertilizer (N0), inorganic N fertilizer (IF), organic N fertilizer (OF), organic N fertilizer + inorganic N fertilizer (OFIF), and slow-release N fertilizer + inorganic N fertilizer (SRIF)] and two tillage treatments (NT and CT) using a split-plot randomized complete block design with three replications. The N fertilizer sources were used as the main plots and tillage practices as the sub-plots. Each plot was 40 m^2^ (5 m × 8 m) in area. Plastic films were inserted into 40 cm depth covered ridges (40 cm wide and 40 cm high) between the plots for preventing the movement of water and fertilizer. To further prevent the transferring of water and fertilizer, border rows with width of 1 m were planted between treatments.

Middle-season rice was direct-seeded on June 3rd in each year, and the harvest was conducted in early October. Throughout the whole rice season, fertilizers were applied at the rates of 180 kg N ha^-1^, 90 kg P_2_O_5_ ha^-1^, and 180 kg K_2_O ha^-1^ for N fertilizer treatments. Both P (single super-phosphate) and K (potassium chloride) fertilizers were used as basal fertilizers only at the seedling stage, and the N fertilizers were applied in four split doses: at 50% as basal fertilizer, 20% as tillering fertilizer, 12% as jointing fertilizer and 18% as earing fertilizer under IF, OFIF and SRIF treatments. Conventional urea (46%) was used as the topdressing N. For OF treatment, 3082 kg ha^-1^ rape seed cakes (equal to 180 kg N ha^-1^) were used in a single dose at only the seedling stage. For OFIF and SRIF treatments, 925 kg ha^-1^ rape seed cakes (equal to 54 kg N ha^-1^) and 616 kg ha^-1^ slow release fertilizers (equal to 90 kg N ha^-1^) were applied as basal fertilizer, respectively. The details of fertilizer management were described by Zhang et al. (2016).

The fertilizers were spread under NT subplots in which the soil was not disturbed. For CT subplots, the basal fertilizers were applied on the soil surface, and then the soil was plowed to 20 cm depth with a spade and harrowed by a multi-passes of chisel rake subsequently. The water depth of the plots was maintained at the depth of 8 cm during the rice growing season except for the tillering and maturing stages. Herbicides (36% glyphosate at 3 L ha^-1^) and pesticides (20% chlorantraniliprole at 150 mL ha^-1^ and 3% emamectin benzoate at 450 mL ha^-1^) were used to control weeds and pests during rice growing seasons when needed.

### Measurement of NH_3_ Volatilization

The NH_3_ volatilization was measured by the ventilation method immediately after the mid-season rice was directly seeded (Wang et al., 2004; Jantalia et al., 2012). The detailed measurement was described by Liu et al. (2015). During the rice growing seasons of 2013 and 2014, the NH_3_ flux was measured 22 times in each year. Cumulative NH_3_ loss in each plot throughout the whole season was computed according to Liu et al. (2015).

### Rice Plant Sampling and Analysis

To measure the rice grain yield, three frames (1 m × 1 m) were harvested in each plot. The grains were adjusted to the moisture content of 14%. Yield components were investigated from 12 hills sampled from the three harvested frames. The detailed measurement of productive panicle number per m^2^, grain number per panicle, grain filling percentage, and 1000-grain weight was as described by Liu et al. (2015). Moreover, 10 hills in every plot were divided to panicle and straw, oven-dried at 80°C and weighed. The dried tissues were ground to determine the N concentrations by FIAstar5000 continuous flow injection analysis. N uptake was calculated as the product of N concentration and dry matter.

### Data Analysis

The methods as described by Deng et al. (2014) and Liu et al. (2015) were used to compute N recovery efficiency (NRE), N agronomic efficiency (NAE), and N partial factor productivity (NFP). N loss rate through NH_3_ emission was calculated as the ratio of cumulative NH_3_ volatilization to applied amount of N.

Two-way ANOVA with SPSS 12.0 analytical software package was used to determine the effects of N sources and tillage practices on NH_3_ flux, NUE, and grain yield. The least significant difference (LSD) test at the 0.05 or 0.01 probability level was conducted to compare the difference in the means between treatments.

## Results

### NH_3_ Fluxes

**FIGURE 1:**
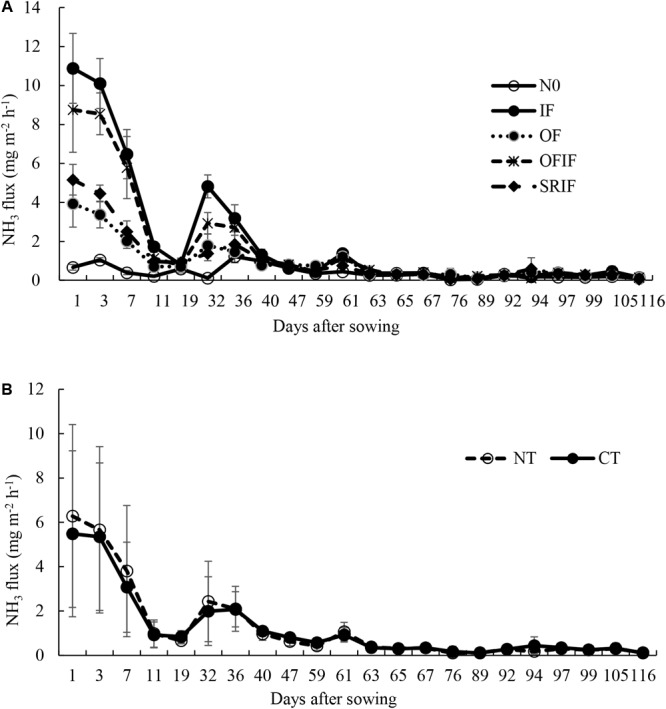
Changes in NH_3_ fluxes from different N fertilizer **(A)** and tillage practice **(B)** treatments during 2013 rice growing season. The arrows indicate N fertilization. N0, no N fertilizer; IF, inorganic N fertilizer; OF, organic N fertilizer; SRIF, slow-release N fertilizer combined with inorganic N fertilizer; OFIF, organic N fertilizer combined with inorganic N fertilizer; NT, no-tillage; CT, conventional intensive tillage.

**FIGURE 2:**
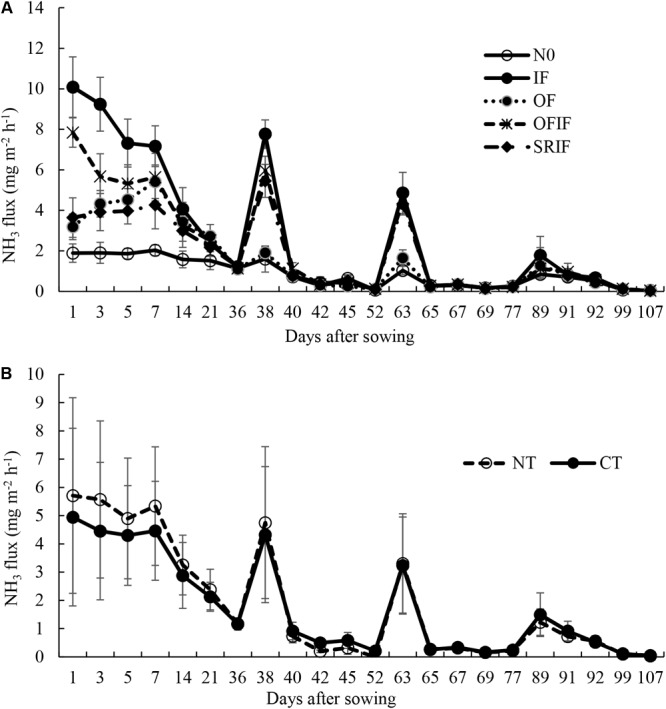
Changes in NH_3_ fluxes from different N fertilizer **(A)** and tillage practice **(B)** treatments during 2014 rice growing season. The arrows indicate N fertilization. N0, no N fertilizer; IF, inorganic N fertilizer; OF, organic N fertilizer; SRIF, slow-release N fertilizer combined with inorganic N fertilizer; OFIF, organic N fertilizer combined with inorganic N fertilizer; NT, no-tillage; CT, conventional intensive tillage.

Seasonal changes of NH_3_ fluxes under different treatments are shown in **Figures 1, 2**. N fertilization significantly increased NH_3_ fluxes, and peaks were observed 1–3 days after each N fertilization. The fluxes under tillage treatments ranged from 0.10 mg m^-2^ h^-1^ to 6.28 mg m^-2^ h^-1^ in 2013, and from 0.003 mg m^-2^ h^-1^ to 5.58 mg m^-2^ h^-1^ in 2014. Moreover, the fluxes under N fertilizer treatments fell within the range of 0.03 mg m^-2^ h^-1^ – 10.89 mg m^-2^ h^-1^ in 2013, and of 0.03 mg m^-2^ h^-1^ – 10.09 mg m^-2^ h^-1^ in 2014.

N fertilizer sources significantly affected cumulative NH_3_ volatilization (**Table 2**). N fertilization remarkably increased the volatilization compared with N0. The volatilization under IF, OF, OFIF, and SRIF treatments were 4.19, 2.13, 3.42, and 2.23 fold in 2013, and 2.49, 1.68, 2.08, and 1.85 fold in 2014 relative to that in N0 treatment, respectively. Tillage practices had no effect on the volatilization throughout the whole seasons. The volatilization from basal fertilizer was obviously different between NT and CT, where NT significantly increased the volatilization by 10–14% in both years compared with CT. Moreover, the volatilization from basal fertilizer accounted for 50–69% in 2013 and 53–76% in 2014 of total volatilization under N fertilizer treatments. Significant interactive effects of N source and tillage practice on the volatilization was only observed in 2013.

**Table 2:** Cumulative NH_3_ volatilization (g m^-2^) at different stages of N application under different treatments.

Treatments	2013					2014				
	Basal	Tillering	Jointing	Earing	Total	Basal	Tillering	Jointing	Earing	Total
N0	0.33 ± 0.05	0.45 ± 0.09	0.11 ± 0.01	0.10 ± 0.01	0.99 ± 0.10	1.32 ± 0.15	0.38 ± 0.06	0.25 ± 0.02	0.13 ± 0.02	2.08 ± 0.15
IF	2.81 ± 0.28	0.92 ± 0.12	0.22 ± 0.07	0.19 ± 0.06	4.14 ± 0.33	3.34 ± 0.30	1.18 ± 0.13	0.48 ± 0.05	0.17 ± 0.04	5.16 ± 0.32
OF	1.09 ± 0.10	0.62 ± 0.03	0.25 ± 0.09	0.15 ± 0.05	2.11 ± 0.15	2.58 ± 0.38	0.45 ± 0.08	0.32 ± 0.09	0.14 ± 0.02	3.49 ± 0.33
NFIF	2.19 ± 0.13	0.79 ± 0.07	0.22 ± 0.06	0.17 ± 0.04	3.38 ± 0.18	2.74 ± 0.29	1.06 ± 0.06	0.37 ± 0.04	0.16 ± 0.01	4.33 ± 0.22
SRIF	1.27 ± 0.07	0.62 ± 0.13	0.15 ± 0.02	0.16 ± 0.05	2.20 ± 0.13	2.23 ± 0.31	1.00 ± 0.11	0.46 ± 0.14	0.16 ± 0.04	3.86 ± 0.32
NT	1.61 ± 0.97	0.65 ± 0.23	0.19 ± 0.06	0.37 ± 0.06	0.15 ± 1.24	2.60 ± 0.77	0.78 ± 0.36	0.36 ± 0.09	0.15 ± 0.03	3.89 ± 1.16
CT	1.47 ± 0.85	0.71 ± 0.14	0.19 ± 0.09	0.41 ± 0.05	0.16 ± 1.05	2.28 ± 0.67	0.85 ± 0.34	0.40 ± 0.13	0.16 ± 0.03	3.69 ± 0.32
*F*-value										
N source	446.55^∗∗^	28.98^∗∗^	4.76^∗∗^	3.23^∗^	355.26^∗∗^	58.34^∗∗^	97.10^∗∗^	7.87^∗∗^	2.26^ns^	112.22^∗∗^
Tillage practice	12.25^∗∗^	3.62^ns^	0.10^ns^	0.70^ns^	1.31^ns^	14.04^∗∗^	4.76^∗^	1.81^ns^	1.78^ns^	4.30^ns^
N source × Tillage practice	3.18^∗^	2.59^ns^	0.40^ns^	0.83^ns^	4.03^∗^	0.95^ns^	0.31^ns^	0.12^ns^	0.70^ns^	0.91^ns^

*Different letters between N fertilizer treatments under the same tillage practice indicate significant differences at the 5% level. N0, no N fertilizer; IF, inorganic N fertilizer; OF, organic N fertilizer; SRIF, slow-release N fertilizer combined with inorganic N fertilizer; OFIF, organic N fertilizer combined with inorganic N fertilizer; NT, no-tillage; CT, conventional intensive tillage. “^∗^” and “^∗∗^” mean *P* < 0.05 and *P* < 0.01, respectively; ns, not significant.*

### Grain Yields and Yield Components

N fertilization significantly enhanced the grain yield due to the increase of productive panicle number, grain number per panicle, and grain filling percentage (**Table 3**). The grain yield under IF, OF, OFIF, SRIF treatments was 1.23, 1.15, 1.30, and 1.42 fold in 2013, and 1.17, 1.10, 1.30, and 1.28 fold in 2014 compared with that under N0 treatment, respectively. Moreover, tillage practices did not affect rice grain yield and its components. No significant interactive effects of N sources and tillage practices on grain yield were observed in each year, while there were significantly interactive effects of N sources and tillage practices on grain number per panicle and grain filling percentage in 2013 and on grain filling percentage in 2014.

**Table 3:** Grain yields and yield components under different treatments.

Treatments	2013					2014				
	Productive Panicle(10^-2^)	Grain number per panicle	Grain filling percentage (%)	1000-grain weight (g)	Grain yield (kg ha^-1^)	Productive panicle (10^-2^)	Grain number per panicle	Grain filling percentage (%)	1000-grain weight (g)	Grain yield (kg ha^-1^)
N0	217.12 ± 10.31	152.85 ± 7.23	0.72 ± 0.01	26.05 ± 0.60	6474.35 ± 168.64	213.99 ± 9.58	174.77 ± 7.76	74.40 ± 1.14	25.19 ± 0.51	7491.41 ± 135.50
IF	261.94 ± 9.99	175.52 ± 6.17	0.81 ± 0.02	27.60 ± 0.65	7957.60 ± 274.83	232.92 ± 6.75	206.28 ± 14.09	77.59 ± 2.26	24.93 ± 0.16	8798.62 ± 177.88
OF	261.12 ± 15.65	171.99 ± 10.85	0.74 ± 0.01	25.52 ± 0.23	7440.32 ± 255.85	237.24 ± 12.50	193.93 ± 14.72	72.61 ± 6.99	24.77 ± 0.34	8261.00 ± 124.66
OFIF	274.34 ± 13.35	167.42 ± 3.35	0.80 ± 0.01	27.11 ± 0.71	8417.22 ± 172.88	245.47 ± 11.83	202.74 ± 7.82	79.55 ± 4.03	25.28 ± 0.36	9766.00 ± 630.58
SRIF	288.23 ± 15.17	181.62 ± 13.37	0.76 ± 0.01	26.52 ± 0.80	9208.55 ± 473.41	293.83 ± 17.81	194.73 ± 7.73	82.38 ± 1.99	25.73 ± 0.32	9569.00 ± 243.08
NT	259.69 ± 29.33	170.44 ± 10.31	0.77 ± 0.03	26.70 ± 1.01	7959.04 ± 980.43	247.33 ± 30.10	194.13 ± 16.08	77.17 ± 6.60	25.30 ± 0.52	8776.18 ± 966.56
CT	261.40 ± 25.77	169.31 ± 15.39	0.77 ± 0.05	26.42 ± 0.90	7840.17 ± 996.97	242.06 ± 29.48	194.85 ± 14.48	77.44 ± 3.10	25.06 ± 0.32	8778.23 ± 873.46
*F*-value										
N source	21.13^∗∗^	21.39^∗∗^	137.98^∗∗^	10.41^∗∗^	65.90^∗∗^	37.23^∗∗^	6.51^∗∗^	9.08^∗∗^	6.58^∗∗^	43.07^∗∗^
Tillage practice	0.11^ns^	0.29^ns^	0.38^ns^	1.42^ns^	1.10^ns^	1.45^ns^	0.03^ns^	0.06^ns^	3.75^ns^	0.00^ns^
N source × Tillage practice	0.29^ns^	10.01^∗∗^	21.47^∗∗^	0.90^ns^	0.23^ns^	1.19^ns^	0.42^ns^	4.30^∗^	0.54^ns^	0.32^ns^

*Different letters between N fertilizer treatments under the same tillage practice indicate significant differences at the 5% level. N0, no N fertilizer; IF, inorganic N fertilizer; OF, organic N fertilizer; SRIF, slow-release N fertilizer combined with inorganic N fertilizer; OFIF, organic N fertilizer combined with inorganic N fertilizer; NT, no-tillage; CT, conventional intensive tillage. “^∗^” and “^∗∗^” mean *P* < 0.05 and *P* < 0.01, respectively; ns, not significant.*

### NUE

N sources had obvious influence on NUE (**Table 4**). In general, OF treatment resulted in the lowest NRE, NAE and NFP among all N fertilizer treatments, while SRIF and OFIF treatments led to higher NRE, NAE and NFP than IF treatment. OFIF treatments increased the NRE, NAE and NFP by 11–42%, 31–75%, and 6–11%, and SRIF treatments increased the NRE, NAE and NFP by 58–77%, 59–84%, and 9–16%, compared with IF treatments, respectively. No interactive effects of N sources and tillage practices on NUE were observed.

**Table 4:** Nitrogen use efficiency from different treatments.

Treatments	2013			2014		
	NRE (%)	NAE (kgkg^-1^)	NFP (kgkg^-1^)	NRE (%)	NAE (kgkg^-1^)	NFP (kgkg^-1^)
N0	–	–	–	–	–	–
IF	26.63 ± 2.84	8.24 ± 1.61	44.21 ± 1.53	37.45 ± 4.34	7.26 ± 1.29	48.88 ± 0.99
OF	22.81 ± 2.80	5.37 ± 0.88	41.34 ± 1.42	23.51 ± 2.57	4.28 ± 1.15	45.89 ± 0.69
OFIF	37.76 ± 3.48	10.79 ± 0.65	46.76 ± 0.96	41.57 ± 4.17	12.64 ± 3.95	54.26 ± 3.50
SRIF	47.10 ± 2.43	15.19 ± 2.83	51.76 ± 2.63	59.24 ± 3.93	11.54 ± 1.19	53.16 ± 1.35
NT	32.80 ± 10.19	10.03 ± 3.88	46.22 ± 3.95	39.19 ± 12.84	9.31 ± 4.30	50.62 ± 4.21
CT	34.35 ± 10.44	9.77 ± 4.32	45.51 ± 4.25	41.69 ± 14.60	8.55 ± 3.85	50.48 ± 3.73
*F*-value						
N source	83.44^∗∗^	29.76^∗∗^	30.14^∗∗^	100.39^∗∗^	15.57^∗∗^	19.59^∗∗^
Tillage practice	1.64^ns^	0.110^ns^	0.88^ns^	2.90^ns^	0.60^ns^	0.03^ns^
N source × Tillage practice	0.60^ns^	0.25^ns^	0.25^ns^	1.20^ns^	0.24^ns^	0.30^ns^

*Different letters between N fertilizer treatments under the same tillage practice indicate significant differences at the 5% level. N0, no N fertilizer; IF, inorganic N fertilizer; OF, organic N fertilizer; SRIF, slow-release N fertilizer combined with inorganic N fertilizer; OFIF, organic N fertilizer combined with inorganic N fertilizer; CT, conventional intensive tillage; NRE, N recovery efficiency; NAE, N agronomic efficiency; NFP, N partial factor productivity; NT, no-tillage; CT, conventional intensive tillage. “^∗∗^” means *P* < 0.01; ns, not significant.*

## Discussion

This study investigated the effects of N sources and tillage practices on NH_3_ volatilization, grain yield and NUE from paddy fields in central China. The results part supported our hypotheses that N sources had significant effects on NH_3_ volatilization, grain yield and NUE, and SRIF treatment had the second-lowest NH_3_ volatilization and the highest grain yield and NUE among N fertilizer treatments. However, tillage practices only influenced NH_3_ volatilization at the early stage of rice under N fertilized conditions, but did not affected grain yield and NUE.

### NH_3_ Volatilization

NH_3_ flux peaks observed 1–3 days after each N fertilizer treatment (**Figures 1, 2**) may be attributed to the enzymatic hydrolysis of the applied N (Zhang et al., 2011; Shang et al., 2014). Enhancement of NH_3_ volatilization caused by N fertilization has been reported in numerous studies (Zhang et al., 2011; Liu et al., 2015; Huang et al., 2016).

In the present study, the cumulative NH_3_ volatilization under IF treatment was estimated to be 41.4–51.6 kg ha^-1^, which is similar to the results reported by Liu et al. (2015) in this region. The cumulative NH_3_ volatilization accounting for 50–76% of total NH_3_ volatilization occurred in basal fertilizer under N fertilizer treatments in both years (**Table 2**). Similar results were observed by Xu et al. (2012), who reported that the cumulative NH_3_ volatilization from basal and tillering fertilizer (about 1 month) accounted for more than 80% of the total NH_3_ volatilization from rice fields in the Tai-lake region of China. The high volatilization in this stage may be due to the application of relatively more N fertilizers and high temperatures in this stage (**Table 1**). Moreover, a previous study indicated that dense canopy may act as a sink of NH_3_ in more vigorous stages (Bash et al., 2010). Thus, the climate conditions in the sparse canopy at the early stages of rice growth may facilitate NH_3_ emission (Cao and Yin, 2015; Zhao et al., 2015).

In this study, compared with IF treatment, the other three N fertilizer treatments significantly decreased NH_3_ volatilization (**Table 2**), suggesting that the application of organic N or slow-release N fertilizers is an effective strategy for mitigating NH_3_ emission from paddy fields. The results are consistent with those reported by Xu et al. (2013), Ye et al. (2013), Huang et al. (2016), and Ke et al. (2017). Moreover, the OF treatment resulted in the lowest NH_3_ volatilization among all N fertilizer treatments, which may be due to the relatively low availability of N from the decomposition of rape seed cake (Bayu et al., 2006). NH_4_^+^ released from the mineralization of the rape seed cake can be partly immobilized by microbes and the soil (Heal et al., 1997; Yang et al., 2015), which thereby reduces NH_4_^+^ concentration in soil and then decreases the NH_3_ volatilization. Compared with OFIF treatment, SRIF treatment decreased the NH_3_ volatilization (**Table 2**) possibly due to a synchronization of N release with rice requirement (Ke et al., 2017). The prolonged release of fertilizer N matches the requirement of rice, and reduces soil NH_4_^+^ concentrations and NH_3_ volatilization subsequently (**Figures 1, 2** and **Table 2**).

In this study, higher NH_3_ volatilization under N fertilized conditions from NT at the early stage of rice growth was higher than that from CT (**Table 2**). Similar results were reported by Zhang et al. (2011). Greater urease activities in soil surface under NT than CT (Zhang et al., 2011) may result in higher concentration of NH_4_^+^ in the soil and floodwater from hydrolyzed fertilizer N, which thereby promotes NH_3_ emission under NT. Moreover, the contact of fertilizer particles and the soil may be reduced by the residues retained in NT soil surface, which means reduced adsorption of NH_4_^+^ from hydrolyzed fertilizer N by soil particles under NT. However, dense canopy may act as a sink of NH_3_ in the middle and later stages of rice growth (Bash et al., 2010). Therefore, promoting effects of NT on NH_3_ volatilization was only recorded in the early stage of rice growth in this study (**Table 2**). As Rochette et al. (2009) reported, fraction of fertilizer N may diffuse into the shallow cracks under CT, which may result in lower NH_3_ volatilization at the early stage of crop growth under CT than under NT.

### Grain Yield

The lowest yield was recorded under OF treatment, while the highest was observed under OFIF and SRIF treatments (**Table 3**). It has been reported that the application of organic matter alone may not be enough to sustain crop yield due to its relatively low nutrient supply (Bayu et al., 2006), which is in agreement with the results reported by Yang et al. (2015), Wei et al. (2016), and Zhang et al. (2017). These results demonstrate that it is impossible to increase the grain yield to meet the food demand in the world through establishing a rice production system that depends exclusively on organic matter (Seufert et al., 2012). However, it was noted that the use of organic fertilizer alone can substantially increase the yield if sufficiently large quantities are applied (Wei et al., 2016). For example, Lu et al. (2012) found that the application of 270 kg N ha^-1^ slurry can bring about a rice grain yield similar to that results from the application of 270 kg N ha^-1^ urea. We found that the application of organic N fertilizer + inorganic N fertilizer (OFIF) resulted in a higher grain yield than the application of inorganic N fertilizer only (**Table 3**), which may be due to the improvement of nutrient efficiency and organic matter impacts (Han et al., 2004; Wei et al., 2016). Wei et al. (2016) performed a comprehensive review based on 32 long-term experiments in China, and reported the positive effects of the combination of organic and inorganic fertilizers on rice grain yield. However, Zhang et al. (2017) found that amending inorganic fertilizer with anaerobically digested pig slurry had no significant effects on rice grain yield. This discrepancy may be attributed to different types of organic fertilizers and the ratio of organic and inorganic fertilizers used (Wei et al., 2016). Slow-release N fertilizer can release N into the soil that can closely match the N demand in different growing stages of crop, which has been widely implemented in China to improve crop production and mitigate environmental problems caused by the application of inorganic fertilizers (Yang et al., 2015; Zheng et al., 2016; Ke et al., 2017). In this study, the substitution of half of inorganic N fertilizer by slow-release N fertilizer resulted in a higher grain yield relative to inorganic N fertilizer (**Table 3**). The N released from slow-release N fertilizer at the early and middle stages of rice growth is relatively low, which may result in N supply deficiency at the stages (Ke et al., 2017); thus, topdressing N at the tillering and jointing stages may better satisfy the N demand at different growing stages of rice in this study. Chen et al. (2010) and Ke et al. (2017) also reported that a mixture of inorganic and slow-release N fertilizers could increase grain yield.

In the present study, the grain yield was increased under N fertilization due to the increase of productive panicle number, grain number per panicle, and grain filling percentage (**Table 3**), which is basically consistent with the previously reported results (Li et al., 2017).

Yield is an important indicator to assess the response of crop to tillage practices. In this study, no significant effects of tillage practices on grain yield were observed (**Table 3**). The effects of NT practice on rice grain yield can be promoting (Gao et al., 2004), decreasing (Gathala et al., 2011), and no effect (Zhang et al., 2011, 2016) compared with CT practice. For example, Gao et al. (2004) reported that NT significantly increased rice yield in eastern China compared with CT because of the improvement of paddy soil physical and chemical properties. Sharma et al. (2005) reported the reduction of rice yields in rice-based systems under NT in northern India. In the northwestern Himalayan region, NT did not affect rice yield compared with CT (Panday et al., 2008). The variables might be related to soil properties (e.g., texture and pH), climates (e.g., temperature and light) and field management practices (e.g., N application rate, planting method, crop rotation, residue management, and the duration of NT use) (Xie et al., 2007; Gathala et al., 2011; Huang M. et al., 2012). Moreover, the yields varied between the 2 years in this study (**Table 3**), which might result from the year-specific climate (**Table 1**).

### NUE

N source significantly affected NUE of rice, and OF treatment resulted in the lowest NUE among four N fertilizer treatments (**Table 4**). Bayu et al. (2006) proposed that the application of organic materials alone may not be enough to maintain crop production due to the limited availability and relatively low nutrient content of organic materials. Moreover, it is commonly believed that the combination of organic and chemical N fertilizers can reduce N losses by converting inorganic N into organic forms, and thus can enhance the efficiency of the fertilizers compared with the application of inorganic N fertilizer alone (Yang et al., 2015). The combination could improve the nutrient uptake efficiency of crops (Han et al., 2004). Thus, we found higher NUE under OFIF than under IF in this study (**Table 4**). Slow-release N fertilizer has been reported to decrease N losses through denitrification, NH_3_ volatilization (**Table 2**), N leaching and N runoff because N release of the fertilizer can closely match the N demand at the later stages of rice growing (Timilsena et al., 2015; Ke et al., 2017). Therefore, although half of inorganic N fertilizer was replaced by slow-release N fertilizer in this study, SRIF treatment resulted in higher NUE compared with IF treatment. Similar result was reported by Ke et al. (2017), who observed that the combination of organic and inorganic N fertilizers (83.3%:16.7%) resulted in higher NUE than the application of inorganic N alone due to the relatively uniform N release from slow-release N fertilizer and the synchronization of the N release with the N requirement of rice.

Although it has been reported that NT promotes N losses through NH_3_ volatilization, N leaching, and N runoff (Zhang et al., 2011; Liang et al., 2016), the combination of NT with retained residues could help to reduce the negative effects of NT on the N losses in paddy fields due to the improvement of properties, fertility and microbial activities in the soil, which can provide rice with sufficient N sources (Huang J. et al., 2012). In the present study, the previous crop resides were retained in the field, and thus no significant effect of tillage practices on NUE was observed (**Table 4**). The result was inconsistent with the result based on meta-analysis (Liang et al., 2016) that NT overall decreased N uptake and NUE. The discrepancy may be attributed to the differences in agricultural management practices, climate and soil property and the duration of NT (Liang et al., 2016).

Our previous study has reported that SRIF plus NT showed the lowest global warming potential and greenhouse gas intensity among all treatments (Zhang et al., 2016). Therefore, from this study, the SRIF plus NT treatment may be recommended as a sustainable strategy to reduce greenhouse gas and NH_3_ emissions, and increase grain yield and NUE in central China.

## Conclusion

N sources remarkably affected NH_3_ volatilization, NUE, and grain yield; while tillage practices had significant effects on NH_3_ volatilization, but had no effects on grain yield and NUE. SRIF treatment resulted in relatively low NH_3_ volatilization and high grain yield and NUE. Our results suggest that the combination of SRIF and NT is an economic and environmental strategy for mitigating greenhouse gas and NH_3_ emissions, improving NUE, and increasing rice yields in central China.

## Author Contributions

CL and CC designed the research. TL, JH, and KC performed the experiments. TL analyzed the data and wrote the manuscript. All of the authors read and approved the final manuscript.

## Conflict of Interest Statement

The authors declare that the research was conducted in the absence of any commercial or financial relationships that could be construed as a potential conflict of interest.
